# Antennal transcriptome analysis reveals sensory receptors potentially associated with host detection in the livestock pest *Lucilia cuprina*

**DOI:** 10.1186/s13071-024-06391-6

**Published:** 2024-07-18

**Authors:** Juan P. Wulff, Paul V. Hickner, David W. Watson, Steven S. Denning, Esther J. Belikoff, Maxwell J. Scott

**Affiliations:** 1 Department of Entomology and Plant Pathology, North Carolina State University, Raleigh, NC 27695 USA; 2 United States Department of Agriculture, Agricultural Research Service, Knipling-Bushland U.S. Livestock Insects Research Laboratory, 2700 Fredericksburg Road, Kerrville, TX 78028-9184 USA

**Keywords:** Myiasis, Blowfly livestock pest, Australian sheep blowfly, Host seeking, RNA-Seq, DESeq2, Chemoreceptor, Mechanoreceptor, Antennae

## Abstract

**Background:**

*Lucilia cuprina* (Wiedemann, 1830) (Diptera: Calliphoridae) is the main causative agent of flystrike of sheep in Australia and New Zealand. Female flies lay eggs in an open wound or natural orifice, and the developing larvae eat the host’s tissues, a condition called myiasis. To improve our understanding of host-seeking behavior, we quantified gene expression in male and female antennae based on their behavior.

**Methods:**

A spatial olfactometer was used to evaluate the olfactory response of *L. cuprina* mated males and gravid females to fresh or rotting beef. Antennal RNA-Seq analysis was used to identify sensory receptors differentially expressed between groups.

**Results:**

*Lucilia cuprina* females were more attracted to rotten compared to fresh beef (> fivefold increase). However, males and some females did not respond to either type of beef. RNA-Seq analysis was performed on antennae dissected from attracted females, non-attracted females and males. Transcripts encoding sensory receptors from 11 gene families were identified above a threshold (≥ 5 transcript per million) including 49 ATP-binding cassette transporters (ABCs), two ammonium transporters (AMTs), 37 odorant receptors (ORs), 16 ionotropic receptors (IRs), 5 gustatory receptors (GRs), 22 odorant-binding proteins (OBPs), 9 CD36-sensory neuron membrane proteins (CD36/SNMPs), 4 chemosensory proteins (CSPs), 4 myeloid lipid-recognition (ML) and Niemann-Pick C2 disease proteins (ML/NPC2), 2 *pickpocket* receptors (PPKs) and 3 transient receptor potential channels (TRPs). Differential expression analyses identified sex-biased sensory receptors.

**Conclusions:**

We identified sensory receptors that were differentially expressed between the antennae of both sexes and hence may be associated with host detection by female flies. The most promising for future investigations were as follows: an odorant receptor (LcupOR46) which is female-biased in *L. cuprina* and *Cochliomyia hominivorax* Coquerel, 1858; an ABC transporter (ABC G23.1) that was the sole sensory receptor upregulated in the antennae of females attracted to rotting beef compared to non-attracted females; a female-biased ammonia transporter (AMT_Rh50), which was previously associated with ammonium detection in *Drosophila melanogaster* Meigen, 1830. This is the first report suggesting a possible role for ABC transporters in *L. cuprina* olfaction and potentially in other insects.

**Graphical Abstract:**

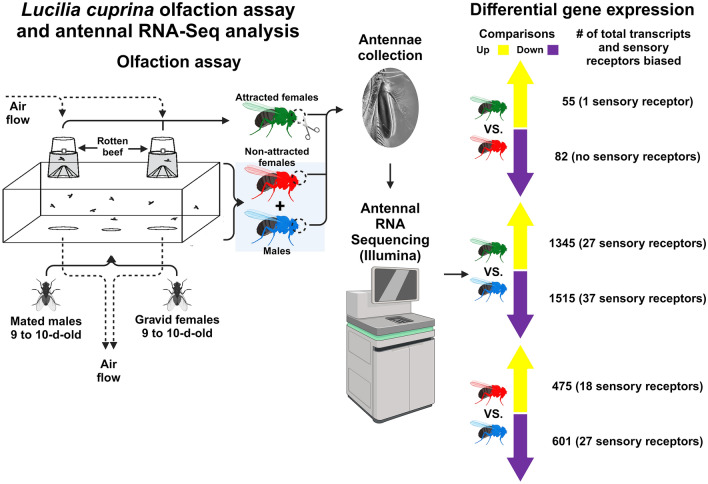

**Supplementary Information:**

The online version contains supplementary material available at 10.1186/s13071-024-06391-6.

## Background

Blowflies (Diptera: Calliphoridae) are key environmental decomposers of organic material making vital nutrients available to other organisms [1]. Calliphoridae, after inclusion of the Rhiniidae with ~ 400 spp [2], includes about 1500 species showing a wide spectrum of feeding specializations [3]. Many of these species are associated with carrion decomposition [4] and have been used in forensic analyses, such as to establish post-mortem intervals [5]. However, not all blowflies are necrophagous, and some represent important livestock ectoparasites, such as *Lucilia cuprina* (Wiedemann, 1830), *Lucilia sericata* (Meigen, 1826) and *Cochliomyia hominivorax* Coquerel, 1858 [6, 7].

The Australian sheep blowfly, *L. cuprina,* is a facultative parasite and a major pest of sheep [6, 8]. That is, they can feed on both carrion and living animals [6]. This species is the main causative agent of sheep flystrike in Australia [9] and New Zealand [10]. Current estimates show that economic losses in Australia reach ~ $175 million annually [11]. The current integrated pest management (IPM) strategies mainly focus on the use of conventional insecticides [12]. However, *L. cuprina* have developed resistance to some insecticides such as organophosphates and benzoylphenyl compounds in Australia and New Zealand [12]. Alternative control strategies such as biological control, vaccines and the sterile insect technique (SIT) have been considered but have not yet been implemented [8]. In line with these approaches, we have developed male-only strains for genetic suppression of *L. cuprina* as an alternative strategy to control this pest [13, 14].

The use of attractive baits associated with traps is an essential element within IPM strategies for control of *L. cuprina* and other blowflies [15–17]. For instance, as part of the *C. hominivorax* SIT eradication program, synthetic baits called *swormlure* were developed from natural screwworm attractants and routinely used to monitor the pest status in the field after mass fly releases [17]. The efficacy of these blends varies over time as flies evolve and adapt to changes in the environment [18]. These formulations have been modified over time from their original composition to obtain a more efficient product and safer for people and the environment [19, 20]. For *L. cuprina*, a similar process was conducted to develop more efficient attractive baits [21].

Fly oviposition behavior is key to understanding flystrike on live animals (hosts) [6, 10]. The females of parasitic species lay their eggs in open wounds or moist tissues on the host [22]. Finding suitable hosts for oviposition is primarily guided by olfactory stimuli [16, 19]. Once a host is selected, females use receptors associated with the labellum, tarsi and ovipositor to select an oviposition site which will provide the larvae with enough food to complete their development [23–25]. In blowflies (as in other insects), chemical compounds from the surrounding environment enter the fly's body through porous sensilla located in the cuticle [26]. Once inside the sensillum, these compounds are transported through the endolymph’ sensillum by odorant-molecule carriers to membrane-bound receptors located on the dendrites of olfactory sensory neurons (OSNs) [26]. These neurons are connected to the central nervous system (CNS), which will integrate the olfactory response and triggers the physiological processes and downstream behaviors [26].

Different sensory receptors have been identified in blowflies, namely, membrane receptors such as odorant receptors (ORs), gustatory receptors (GRs) and ionotropic receptors (IRs) [25, 27–29], and odor-molecule carriers, including chemosensory proteins (CSPs), odorant-binding proteins (OBPs) and CD36-sensory neuron membrane proteins (CD36/SNMPs) [25, 27–30]. However, beyond these descriptive works, there is no functional information on these receptors in Calliphoridae. In other Dipteran species, ORs respond to a variety of volatile organic compounds (VOCs), including pheromones and general odorants [31], whereas GRs sense non-volatile compounds by direct contact through gustatory sensilla, but are also associated with carbon dioxide (CO_2_) detection [32]. Furthermore, IRs are other membrane sensory-related genes associated with taste and olfaction in flies [33]. Functional studies are needed to understand the role of sensory receptors in blowflies. For example, CRISPR/Cas9 was used to knock out the odorant co-receptor (*Orco*) gene in *C. hominivorax* [34]. This work showed that *Orco*-silenced flies presented a decreased response to floral-like and animal host-associated odors, suggesting that the olfaction mediated by odorant receptors is involved in foraging and host-seeking behaviors in *C. hominivorax.*

In addition to the sensory receptors, other proteins could play a role in important behaviors in blowflies such as mating, food-seeking or host detection. For instance, in *Drosophila melanogaster* Meigen, 1830 (Diptera: Drosophilidae), an ATP-binding cassette transporter (ABC) has been associated with the olfactory response [35] and mating [36]. Furthermore, ammonium transporters (AMTs) may act as non-canonical chemoreceptors associated with ammonia sensing in *D. melanogaster* [37, 38]. This is of interest, as ammonia is an important blowfly attractant [39]; consequently, this family of receptors could have a role in *L. cuprina* oviposition behavior. Among other membrane receptors, *pickpocket* receptors (PPKs) were associated with feeding [40] and pheromone detection [41] in *D. melanogaster* and thus could be associated with food-seeking and mating in blowflies. Transient receptor potential channels (TRPs) were also found expressed in the antenna of *D. melanogaster* [42]. These receptors have been associated with sensation of a broad spectrum of stimuli in the same species, such as CO_2_, light, mechanical stimuli, temperature and taste [42]. Regarding CO_2,_ this compound was observed to stimulate oviposition in the blowfly *L. sericata* [16]. The lipid-binding Niemann-Pick C2 disease proteins (ML/NPC2) were recently identified as odorant-molecule carriers in many insect species including *D. melanogaster* [43]. These proteins could also play a role as odor carriers in *L. cuprina*. CD36/SNMP proteins are also associated with olfaction in Diptera [44]. In *D. melanogaster*, SNMP 1 acts as a co-receptor assisting OBPs and ORs in recognizing pheromones [45]. Other members of this family, such as scavenger receptor class B type I (SRB1) and croquemort proteins have been identified in the antenna of *D. melanogaster* with putative roles in chemoreception [44].

*Lucilia cuprina* is an early vertebrate carrion colonizer [16] and an aggressive parasitic blowfly species [9, 12]. The objectives of the present work were: (i) to evaluate the olfactory response of *L. cuprina* adults of both sexes to bovine beef under two different decomposition stages to determine which of both stimuli exerts a stronger attraction to the same species; (ii) to identify sensory receptors expressed in the antenna of *L. cuprina* adults of both sexes; (iii) to identify sex-biased sensory receptors that could potentially be associated with host detection. To complete these aims, a spatial olfactometer was used to separate females that were attracted to rotting meat from males and non-attracted females. RNA was collected from antenna for gene expression analysis using RNA-Seq. Genes including sensory receptors expressed in the antenna of each group were identified, and those that were differentially expressed between the three groups were determined. In summary, a better understanding of the behavioral traits, physiology and genetic background of these fly species is essential to support the development of new technologies to control these pests.

## Methods

### *Lucilia cuprina* rearing conditions

All flies used in the present study were the wildtype (*wt*) *L. cuprina cuprina* LA07 strain reared using protocols described previously [13]. *Lucilia cuprina cuprina* is a subspecies of *L. cuprina* found in North America and elsewhere [46]. Hereafter, we refer to this subspecies as *L. cuprina*. Before running assays, flies were kept under the following conditions: 23 ± 1 °C, 40 ± 10% relative humidity (RH) and a non-controlled photoperiod (~ 13:11 light/dark). The Scott laboratory LA07 colony is currently at ~ 165 generations and was established in 2010 from approximately 300 individuals obtained from Dr. Aaron Tarone (Texas A&M, TX, USA). The LA07 *L. cuprina* colony was established by multiple collections of individuals (~ 300 to 500) from the University of Southern California campus and the Miracle Mile neighborhood in Los Angeles, CA, USA , by Dr. Tarone in 2007.

### Adult olfaction assay

#### Olfactometer and assay conditions

The olfaction assays were carried out using a spatial olfactometer developed previously [47]. The olfactometer included four main independent chambers where the flies were released (Fig. S1). Each main chamber had two smaller collection chambers on top (A and B) where meat samples were placed to attract and trap the flies. The main chambers were located counterclockwise around and on top of a table, keeping the collection container A always on the top left of the main chamber. This arrangement avoided a light and temperature bias between the controls and treatments. Each main chamber corresponded to a biological replicate including two choices represented by collection chambers A and B, corresponding to fresh and rotting meat, respectively.

Ground meat (bovine, 70:30 lean/fat) was purchased at a local supermarket and divided into 25 g aliquots. For the “fresh beef” samples, the meat was immediately frozen and stored at −20 °C. For the rotting beef samples, the meat was kept for 5 days (d) at 30 ºC and 50% RH and then stored at – 20ºC. On the day of the behavior assays, 2 g of previously frozen fresh beef samples was mixed with 1 ml filtered tap water and 1 ml Na-citrated bovine blood (Lampire BL, Cat. 7200806, Pipersville, PA, USA). The blood was kept at −20 ºC in 1-ml aliquots until used. For the “rotting beef” samples, 2 g aliquots were mixed with 2 ml filtered tap water.

To avoid cross odor contamination of the chambers, the collection and main chambers were fed with air from outside the building where the olfactometer was located. Also, after passing through the olfactometer system, air was exhausted from the main chambers to a fume hood using a vacuum system. The airflow was set for 1 cubic foot per minute (cfm). The light inside the chambers was not controlled and ranged between 350 and 400 lx provided by a combination of artificial fluorescent light and natural sunlight and determined using a Benetech v-1010-EN-00 digital lux meter. The room temperature (RT) and RH were 25 ± 1ºC and 40 ± 10%, respectively. In addition, half of the total replicates including both treatments were carried out before noon (~ 9 to 11 a.m.) and the other half after noon (~ 12 to 2 p.m.); then, all were compiled to avoid a daytime bias.

#### Experimental design

The flies used for the assays were mated gravid females (9–10 days old, pre-oviposition) and mated males of the same age. We chose to use gravid females as earlier studies had shown that gravid *L. cuprina* females are more responsive to known attractants than virgin females [16]. Flies were kept in the same building where the assays were carried out from the previous day for habituation. If necessary, CO_2_ was used to put the flies to sleep and sex them the day before the assay, and flies of different sexes were kept in separated bottles. The meat used to test the flies was either “fresh” and/or “rotten” beef under conditions described above.

Three different olfaction assays were completed using flies, samples and the room conditions mentioned above. The first two assays were carried out using either 30 or 50 flies per main chamber; flies were not previously sexed, and a sex ratio of ~ 1:1 was assumed. These two assays were used to compare the response of flies of both sexes to fresh and rotten beef. The total duration for both assays was 120 min (min), with the number of flies per chamber being recorded at 15, 30, 45, 60 and 120 min. At the end of the assay, all flies were sexed to determine the number of males and females collected from chambers A and B and the main chamber.

The third assay was used to collect samples for the antennal RNA-Seq experiment. For this assay a mixture of 35 females and 35 males was used. Flies were sexed and counted to determine the exact number of flies per sex ~ 24 h before running the assay. In this assay only rotten beef was placed in both collection chambers (A and B) to separate attracted from non-attracted flies. To start the assay, 35 flies of each sex were released into each main chamber. Females collected from chambers A and B were considered attracted to the rotten beef, and females and males collected from the main chamber were considered non-attracted to the same stimulus. Immediately after the olfaction assay, flies from each biological replicate were anesthetized by placing the chambers at −20 °C for ~ 10 min. Then, the flies were sexed and placed in separated plastic buckets defining three groups: attracted females (AF), non-attracted females (NF) and males (M). Each bucket was placed on ice to keep the flies anesthetized during the antennae collection. Antennae were removed by using dissection forceps (WPI, Worcester, MA, USA) and then immediately placed in a 1.5-ml Eppendorf tube with 300 µl cold RNAlater^™^ (Thermo-Fisher, Waltham, MA, USA) and stored at −80 °C until use. Each biological replicate was a pool including the antennae of 20 adults (either male or female). Five biological replicates (pools) were completed for AF and M and four for NF.

#### Statistical analysis

Statistical analysis of the adult olfaction assay was completed using GraphPad Prism v9 software (San Diego, CA, USA), and results were plotted using the same software. A Student's multiple *t*-test using the False Discovery Rate (FDR) method for *P* value correction with a Q value of 0.05 (5%) was performed to compare the response of the flies to each stimulus (fresh and rotten beef) across different time points and a single t-test to compare the total number of males or females that responded to each stimulus at the end of the assays. The Shapiro-Wilk and Levene tests were used to determine whether the samples met the assumptions of normality and homoscedasticity, respectively. All results were presented as the mean ± standard error of the mean (SEM).

### RNA-Seq experiment

#### RNA isolation

Prior to RNA extraction, RNAlater^™^ was removed, and each sample was washed twice with 1 ml cold 50% ethanol to remove salt. Subsequently, the antennae were resuspended in 250 µl cold Trizol^™^ reagent (Thermo-Fisher). Samples were immediately disrupted using a Benchmark benchtop homogenizer bead and Benchmark 2.0-ml tubes prefilled with high-impact zirconium beads (Benchmark Scientific, Tempe, AZ, USA). The homogenizer was set to 6.5 m/s, samples were disrupted for 1 min, and then the tubes were placed 1 min on ice. This process was repeated three times for each sample.

Following homogenization, total RNA was extracted using the Zymo Quick-RNA^™^ Microprep kit (Zymo Research, Irvine, CA, USA), according to the manufacturer's specifications, with the exception that two deoxyribonuclease 1 (DNAse1) steps were conducted to completely remove the genomic DNA (gDNA) from the samples as follows: (1) during RNA extraction 30 U of DNAse1 was added to the crude samples followed by 15 min of incubation at RT; (2) after elution of samples in nuclease-free (NF) water, 5 U of DNAse1 and buffer supplied by the manufacturer was added followed by an incubation step as above. After DNAse1 treatments, the RNA Clean & Concentrator^™^-5 kit (Zymo Research, Irvine, CA, USA) was used to clean the samples (following the manufacturer's specifications). The clean total RNA was recovered in 12 µl of NF water and was quantified (1:10 dilution) by fluorometric quantification using a Qubit-4^™^ fluorometer and a high-sensitivity quantification kit (Thermo-Fisher). Samples were stored at −80 °C until used.

#### RNA-Seq assay and data analysis

Library construction and sequencing services were provided by Novogene Inc. (Sacramento, CA, USA). The approach used was Illumina NovaSeq 6000, paired end (PE) 150-bp reads and 50 million reads per sample. For library construction, RNA samples were enriched using oligo(dT) beads (Illumina, San Diego, CA, USA). Subsequently, the mRNA was fragmented randomly using a fragmentation buffer (Illumina) followed by the first-strand cDNA synthesis using mRNA as a template, random hexamer primers, a custom buffer (Illumina), dNTPs (Invitrogen) and DNA polymerase I (Thermo-Fisher). The second strand was synthesized after a RNAse H treatment (Thermo-Fisher). Finally, after the sequence terminal repair, sequencing adaptors (Thermo-Fisher) were ligated. The double-stranded cDNA library was completed through size selection and PCR enrichment.

Geneious Prime^®^ v2023.2.1 (https://www.geneious.com) was used to assess read quality and remove Illumina adaptors. BBDuk v38.88 (https://jgi.doe.gov/data-and-tools/software-tools/bbtools/) was utilized to trim off low-quality bases at the 5′ and 3' ends using the following parameters: kmer length: 27; trim both ends: minimum quality Q30; trim adapters based on paired reads overhangs: minimum overlap 24; discard short reads: minimum length 30 bp. Geneious Prime was used to complete the read mapping and calculate transcript expression and differential transcript expression between groups. Reads were mapped to the reference *L. cuprina* genome, NCBI ID ASM2204524v1, using the default Geneious Prime mapper set at a medium sensitivity. Transcript expression was calculated for all types of annotated RNAs in the reference genome, and ambiguously mapped reads were counted as partial matches. Transcript expression was normalized using transcript per million (TMP) normalization [48]. Expression ranges (low, medium, high) were defined using a method previously used for RNA-Seq data [49, 50] and based on all transcripts expressed in the antenna of *L. cuprina*. Low expression was considered below quartile (Q) 1, medium between Q1 and Q3 and high above Q3. The differential gene expression (DGE) analysis for all transcripts expressed in the antenna of *L. cuprina* was performed using DESeq2 (https://www.bioconductor.org/packages/release/bioc/vignettes/DESeq2/inst/doc/DESeq2.html) and a parametric fit for the dispersion. A similar approach to analyze transcript expression and differential expression was previously used in blowflies [51]. Transcripts expression was plotted using R packages ggplot2 (https://ggplot2.tidyverse.org/) and viridis (https://cran.r-project.org/web/packages/viridis/index.html). Differential transcript expressions and volcano plots were plotted using Microsoft Excel (Redmond, WA, USA). Only transcripts with a TMP expression ≥ 5 were used for downstream analysis of sensory receptors as protein-coding transcripts expressed above a low expression threshold are more likely to be biologically relevant to fly olfaction [52].

#### Chemosensory gene annotations and phylogenetic analysis

Manual annotation of the ORs, GRs, IRs and OBPs was conducted by using BLASTn and tBLASTn analysis of the *L. cuprina* genome assembly and transcriptome using *C. hominivorax* gene models as described previously [28]. BLAST analysis was completed using Geneious Prime^®^ v2023.2.1 (https://www.geneious.com). The *L. cuprina* gene models were evaluated with the aid of multiple protein alignments and gene trees with *D. melanogaster* and *C. hominivorax* chemosensory proteins. Alignments were done using the Muscle alignment tool [53] on the EMBL-EBI web browser [54], except for the IRs, which were aligned using the E-INS-i method in MAFFT v7 [55]. All four alignments were trimmed using the “strict” method in Trimal v1.3 (Trimal) on the Phylemon 2.0 web browser [56].

Phylogenetic analyses were conducted using the IQ-TREE web server (http://iqtree.cibiv.univie.ac.at/) with the auto substitution option and 1000 ultrafast bootstrap replications [57]. Protein sequences of *D. melanogaster* and *C. hominivorax* were used for the phylogenetic analysis; further details about sequences from each receptor gene family are provided in Table S1. Gene tree figures were produced using the interactive Tree of Life (iTOL) v6.8.2 software (https://itol.embl.de/). The ORs were rooted at the odorant co-receptor (Orco), the GRs were rooted at the clade containing the *D. melanogaster* CO_2_ receptors, the IRs were rooted at the Ir25a/Ir8a clade, and OBPs were rooted at the mid-point. If necessary, after manual curation, the LOCI annotations were corrected before running the transcript expression analysis by running a NCBI-Blastn using the CDS sequence of the receptor against the *L. cuprina* genome ASM2204524v1.

#### Gene Ontology enrichment analysis

Gene Ontology (GO) analysis was performed using the online software gProfiler (https://biit.cs.ut.ee/gprofiler/gost). The significance threshold was adjusted following the model Benjamini-Hochberg FDR, and those GO terms with a *P* adjusted value (*Padj*) < 0.05 were considered enriched within a group. Enriched GO terms were plotted using a Manhattan plot provided with the same software.

## Results and discussion

### Adult olfaction assay

**Fig. 1:**
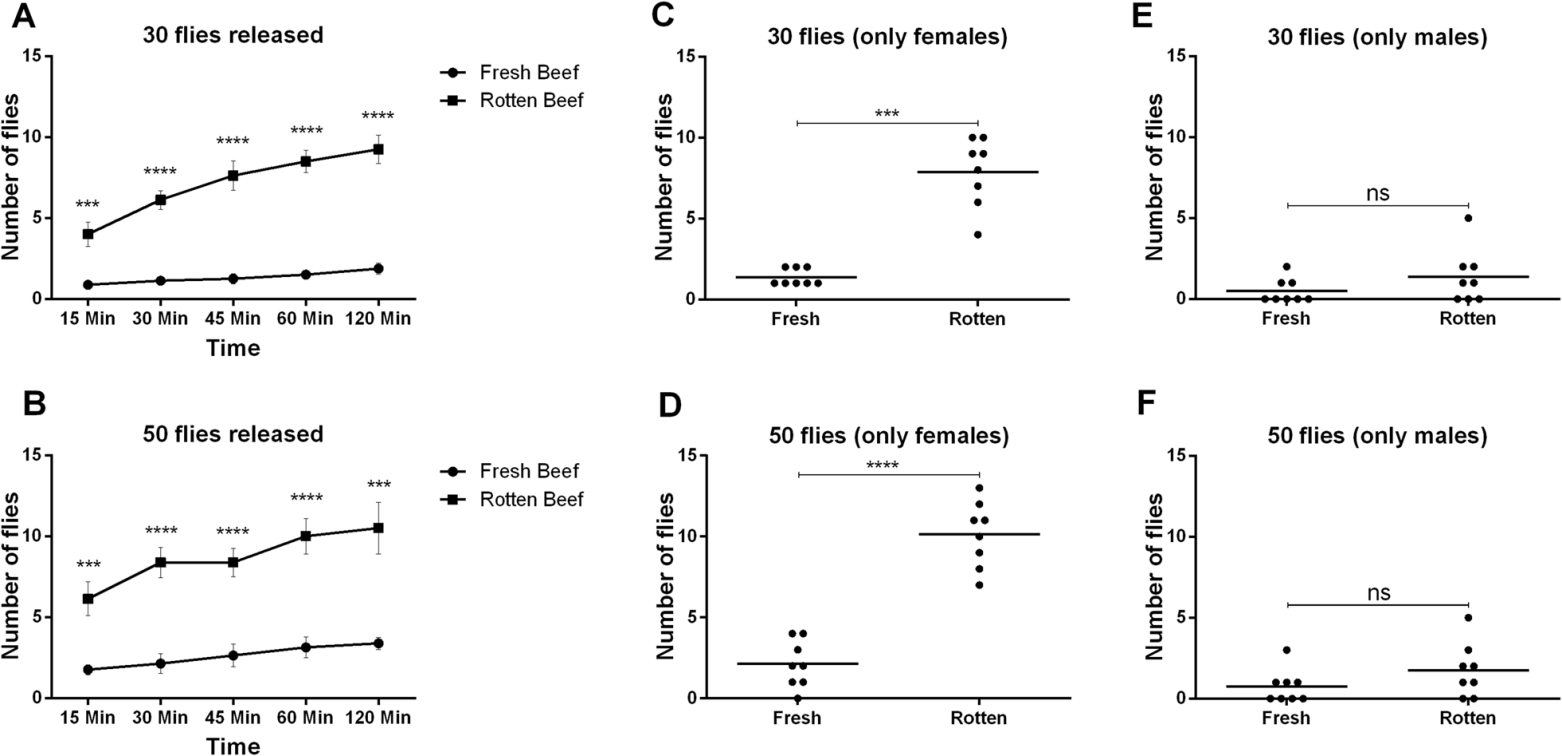
Adult olfaction assay shows *Lucilia cuprina* females are attracted to rotting beef. The adult olfaction assay was initially completed using *L. cuprina* flies of mixed sexes; either a total of 30 (**A**) or 50 (**B**) flies were released into the spatial olfactometer. In both assays, flies were strongly attracted to rotting meat (**A**, **B**). With sex-separated 30 (**C**, **E**) or 50 (**D**, **F**) flies, females were attracted to the rotten beef (**C, D**) but males showed no significant response to either type of beef (**E**, **F**). For more details about the analysis, see Figure S1 and Table S2. A multiple t-test was performed to compare the response of the flies to each stimulus (fresh and rotten beef) across different time points and a single t-test to compare the total number of females or males that responded to each stimulus at the end of the assays. All results were presented as the mean ± standard error of the mean (SEM). *** and ****P < 0.001 and < 0.0001, respectively. *ns* not statistically significant

A spatial olfactometer was used to assess the attraction of *L. cuprina* to fresh or rotting beef (Fig. S1). In this system flies must fly up against an air current to reach the source of the odors. We found that *L cuprina* flies of mixed sexes were more attracted to rotten beef compared to fresh beef (Fig. 1A, B). After determining the sex of the collected flies, it was observed that the females were more attracted (> fivefold increase) to rotten beef (Fig. 1C, D; Table S2). Males showed little response to either fresh or rotten beef (Fig. 1E, F; Table S2). These results are consistent with previous observations that females (virgin and gravid) showed a higher response than males to blowfly attractants [16]. In another blowfly species, *Cochliomyia macellaria* Fabricius, 1830 (Diptera: Calliphoridae), similar responses were observed using flies of mixed sexes and baited with fresh bovine liver [58]. However, Urech et al. [59] did not find any differences in the response of separated *L. cuprina* females and males to fresh liver. Nevertheless, the response of flies varied between different olfactory chambers and days, which suggested this could be due to differences in the volatiles (and/or their concentrations) emitted by different liver samples [53].

The *L. cuprina* gravid females are likely attracted to sulfur-rich chemicals, ammonia and other volatiles emitted from rotten beef [16]. These compounds are released during carrion decomposition [4, 60]. A study that evaluated the response of *C. macellaria* to fresh and rotten livers found that DMDS, DMTS and *p*-cresol were the main attractants for gravid females [61]. Virgin females and males showed a lower response [61]. Furthermore, gravid females of another blowfly, *C. hominivorax*, were more attracted to bovine blood inoculated with bacteria than uninoculated blood [62]. The sulfur-rich chemicals DMDS and DMTS were the main attractants in the inoculated blood. Similarly, the volatiles were emitted by bacteria associated with wounds and feces caught in wool in living sheep that are likely attracting *L. cuprina* females [16, 63–65].

### *Lucilia cuprina* antenna RNA-Seq

### RNA-Seq data analysis and transcript expression

RNA-seq analysis was performed on RNA isolated from the antennae of AF, NF and M. The results are detailed in Table S3 with data analysis in sheets A and B, raw counts in sheet C, transcript expression in D and E, and sex-specific transcripts in F. Read mapping yielded the identification of 24207 transcripts, but only 9847, 9690 and 9647 showed a TMP expression value ≥ 5 in AF, NF and M, respectively (Table S3: E). No significant differences were observed among the three groups regarding transcript levels including all transcripts with a TMP value ≥ 5 (Table S3: E). Antennal RNA-Seq analyses in other Calliphoridae species showed similar results compared to our study. In *C. hominivorax* [29], *Aldrichina grahami* Aldrich, 1930 [25], and *Calliphora stygia* Fabricius, 1781 [27], 10442, 14193 and 16522 non-redundant transcripts were identified, respectively, and in all cases without a minimum expression threshold. However, only 9667 in *A. grahami* and 8709 transcripts in *C. stygia* matched at least one GO term for annotated proteins in other Diptera species [25, 27]. The rest of the transcripts corresponded to orphan genes, pseudogenes and non-coding RNAs, among others [25, 27].

Among transcripts with a TMP value ≥ 5, we found 133 transcripts (85 genes) and 480 transcripts (333 genes), specific for males and females, respectively (Table S3: F). Our results are not in line with the number of sex-specific transcripts observed in the antenna of *A. grahami* [25], where they found 37 and 39 genes specific for males and females, respectively. The differences could be associated with the use of a minimum expression threshold in our analysis and no minimum threshold in the mentioned study. Among sex-biased transcripts, some were potentially associated with olfaction as follows: two esterases, two CYPs, one ABC transporter and one OBP in males and one ABC transporter in females (Table S3: F). However, these sequences were not included in subsequent analyses for sensory receptors because they did not reach the average TMP expression ≥ 5 considering all groups. Transcript expression of sensory receptors with a TMP expression ≥ 5 will be discussed below.

#### Differential gene expression (DGE) analysis

**Fig. 2:**
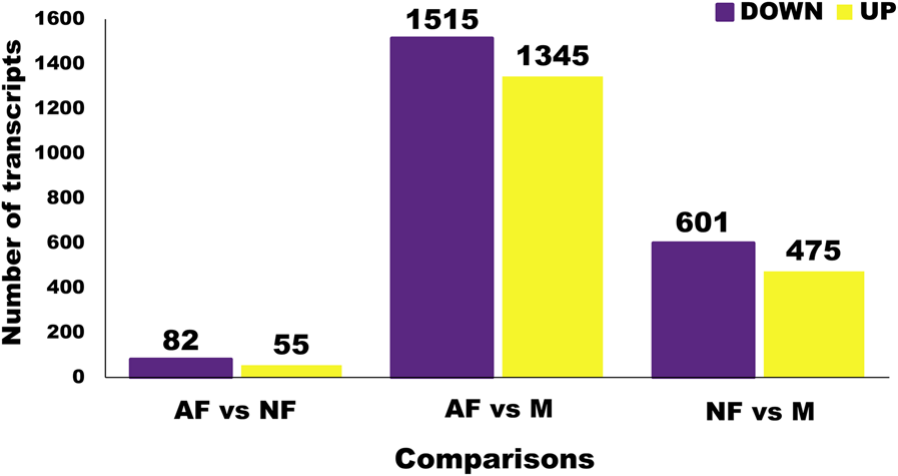
Differential gene expression analysis of all transcripts expressed in the antennae of *Lucilia cuprina*. Total number of transcripts (TMP > 0) down- and upregulated for each group comparison. All differentially expressed transcripts between groups are detailed in Table S4. *AF* attracted female, *NF* non-attracted female, *M* male, *UP* upregulated, *DOWN* downregulated

The total number of differentially expressed (DE) transcripts for the comparisons among the AF, NF and M without a fold-change (FC) cutoff are summarized in Fig. 2. Detailed information about the DE transcripts is presented in Table S4, tabs A–C. The number of biased transcripts between males and females is not comparable to that observed in similar studies in other blowflies because the approaches used were different [25, 27]. However, in those studies and our work, a ~ 1:1 ratio was observed between female- and male-biased transcripts. Among the DE genes, Table S4 details putative odorant-degrading enzymes (ODEs), such as cytochrome P450s (CYP) and esterases, which are of interest as members of these gene families and have been associated with odorant degradation in *D. melanogaster* [66]. For instance, predicted juvenile hormone (JH) esterases were differentially expressed between females (AF and NF) and males (Table S4: B and C, yellow highlighting). These enzymes may also play a role in odorant degradation as a JH esterase was found to be expressed in the antennae of *D. melanogaster* and shown to play a role in reducing the sensitivity to selected odorants [67]. Perhaps not surprisingly, the orthologs of the sex determination genes *transformer* (*tra*) and *doublesex* (*dsx*) were DE, with *tra* being female-biased and *dsx* male-biased. The *L. cuprina* male *tra* transcript does not code for a functional protein and is generally found at lower levels in males than females [68]. *Doublesex* is one of the more strongly male-biased transcripts in *L. cuprina* antennae (Table S4: B and C, yellow highlighted). This suggests that the male DSX protein may directly regulate male-biased genes in the *L. cuprina* antennae. This could be confirmed by using ChIP-seq as done in *D. melanogaster* [69]. Furthermore, *transformer 2* was expressed equally in both sexes, while when *fruitless* (*fru*) transcripts were detected, the levels were well below the 5 TPM threshold (Table S3: D, yellow highlighted).

DE transcripts between groups with a TPM expression ≥ 5 in at least one of the groups are presented in Table S5. Transcripts upregulated within each group vs. the other two groups were placed in separate sheets (Table S5: B-G). There were 32 transcripts from 23 genes that were upregulated in AF vs. both NF and M (Table S5: B), which are thus candidate genes that could play a role in host detection by females. Among the 32 transcripts there was only one sensory receptor, the ABC transporter G23.1, and two CYPs, which are putative ODEs (Table S5: B). Interestingly, ABC G23.1 was the only sensory receptor upregulated between AF vs. NF (Table S4: A). A transcript coding for a gel-forming protein (mucin-5A) was included among the 32 transcripts (Table S5: B) and also upregulated between AF vs. NF (Table S4: A). Mucins are associated with olfaction in vertebrates [70], but in insects, mucins are associated with feeding and reproduction [71, 72]. In *D. melanogaster*, it was observed that the expression of a mucin protein is regulated by an ABC transporter [73]. Furthermore, we found several female-biased mucin transcripts (Table S4: B-C), and two of them only expressed in females (Table S3: F). Glycosylation of mucins is an essential step for the normal function of these proteins [70]. Several transcripts coding for glycosylation enzymes were female-biased (Table S4: B-C). Consequently, these proteins could play a relevant role in *L. cuprina* female olfaction. For other group comparisons, refer to Table S5: C-G.

Given the extensive number of DE sequences between groups below, we will focus only on the predicted sensory receptors. A more detailed analysis of other DE transcripts, such as those coding for ODEs, will be completed in future works.

#### Sensory receptors

**Fig. 3:**
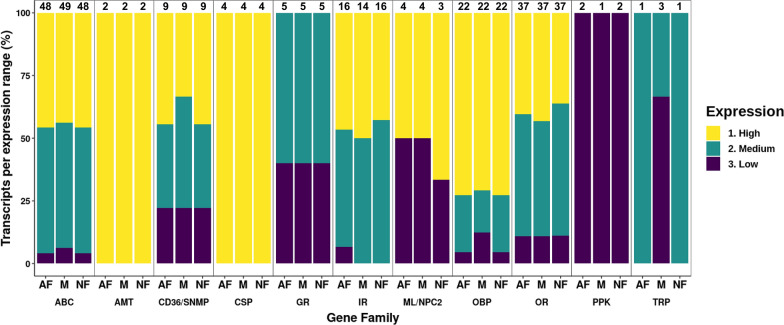
Expression ranges within each sensory receptor gene family and compared group. Expression ranges were defined based on all transcripts expressed in the antennae of *Lucilia cuprina* with low below quartile (Q) 1, medium between Q1 and Q3 and high expression above Q3 (see Materials and Methods). The percentage of transcripts within each expression range for each receptor gene family was calculated based on all transcripts identified for each receptor gene family. The numbers over the bars represent the total number of transcripts within each group. Only transcripts with a TPM value ≥ 5 were used for this analysis. For all transcripts expressed in the antennae of *L. cuprina* associated with each expression range, refer to Table S3: Sheet E. *ABC* ATP-binding cassette transporter, *AMT* ammonium transporter receptor, *CD36/SNMP* CD36 and sensory neuron membrane protein family, *CSP* chemosensory protein, *GR* gustatory receptor, *IR* ionotropic receptor, *OBP* odorant-binding protein, *OR* odorant receptor, *PPK pickpocket* receptor, *TRP* transient potential receptor channel, *AF* attracted female, *NF* non-attracted female, *M* male

Eleven sensory receptor gene families were analyzed (Table S6) and sequences of four of them (OR, IR, GR and OBP) manually curated (Table S7). The number of sequences identified per gene family, considering only transcripts with an average TPM value ≥ 5, was ABC (49); AMT (2); CD36/SNMP (9); CSP (4); GR (5); IR (16); ML/NPC2 (4); OBP (22); OR (37); PPK (2); TRP (3) (Table S6: A–K). The number of sequences identified, and the expression of each receptor cannot be compared with those observed in other blowflies because the experimental design was different, such as the minimum expression threshold used [25, 27, 29]. However, the relative expression between gene families was similar, the soluble carriers being the most highly expressed, namely, CSPs and OBPs, and the lowest expressed were GRs, PPKs and TRPs (Fig. 3 and Table S6: A-K). Also, the abundance in the number of receptors per gene family was similar to other studies on blowfly’ species [25, 27, 29], with ORs > OBPs > IRs > GRs > SNMPs. Other gene families analyzed here were not addressed in those studies.

**Fig. 4:**
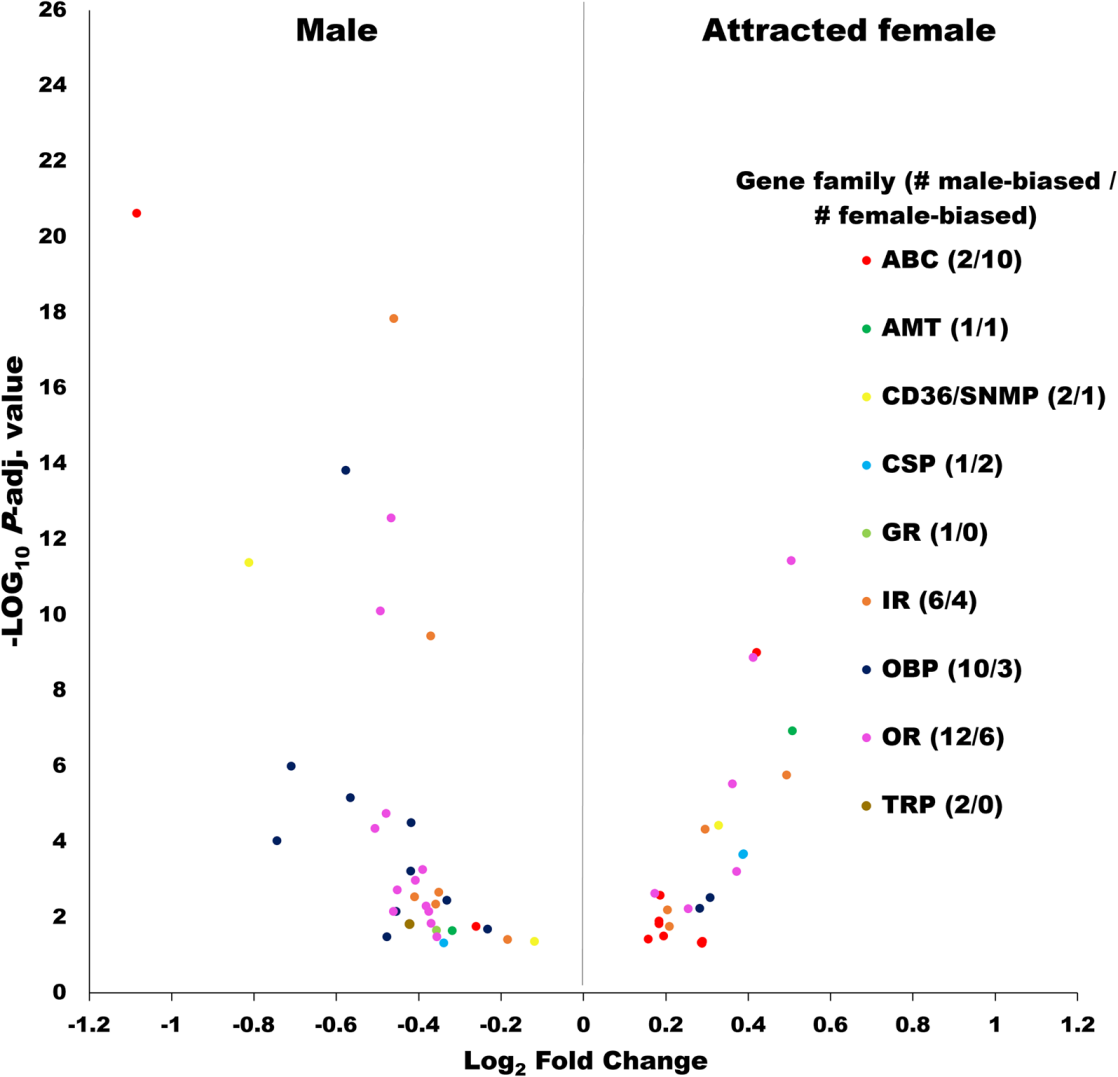
Sensory receptors differentially expressed between males and attracted females. Volcano plot including only transcripts with a TPM expression ≥ 5 (all groups average). The X-axis represents Log_2_ fold-change (Log_2_FC) differences between the contrasted groups, and the Y-axis is the statistical significance as the negative Log_10_ of the *P* adjusted value. The numbers in parentheses correspond to receptors whose antennal expression was male biased (left) or attracted female biased (right). Volcano plots including other group comparisons, and all differentially expressed transcripts are provided in Table S4: D. Abbreviations, same as used in Fig. 3

The number of female- and male-biased receptors is summarized in Fig. 4, and detailed information is provided in Table S6. Regarding the number of biased receptors per gene family, the ABC transporters were the family with the highest number in females (10 upregulated) and the ORs in males (Fig. 4). However, considering FC difference between groups, plus statistical significance, the most relevant were the ORs and OBPs in females and males, respectively (Fig. 4). There were 6 and 12 ORs, biased to females and males, respectively (Fig. 4), with *Orco* among the female-biased ORs. The average TPM expression was > threefold higher for the female-biased ORs (not including *Orco*) compared with the male biased (Table S6). *Orco* is the essential co-receptor for the normal function of all ORs [26]. Our results suggest that the highly expressed female-biased ORs could be driving the expression of *Orco* upward in females.

To our knowledge, only two ORs have been associated with DMDS detection in other invertebrate species, in *Caenorhabditis elegans* Maupas, 1900 (Rhabditida: Rhabditidae) [74], and *Apolygus lucorum* Meyer-Dür, 1843 (Hemiptera: Miridae) [75]. However, we did not find orthologs of these genes in our transcriptome. In addition to ORs, Mahadevan et al. found that a mutation in the cytochrome c oxidase (COX) of *Drosophila busckii* Coquillett, 1901 (Diptera: Drosophilidae) increases the attraction of flies to DMDS and DMTS, but DMTS alone did not induce a significant oviposition preference [76]. Furthermore, flies presented high tolerance to this compound, which is toxic for many species of the same family [76]. Of the 14 subunits that comprise COX, one of them, sub-unit 7A (LOC111688744), was upregulated in the antenna of *L. cuprina* attracted females (Table S4: sheet B, yellow highlighted).

**Fig. 5:**
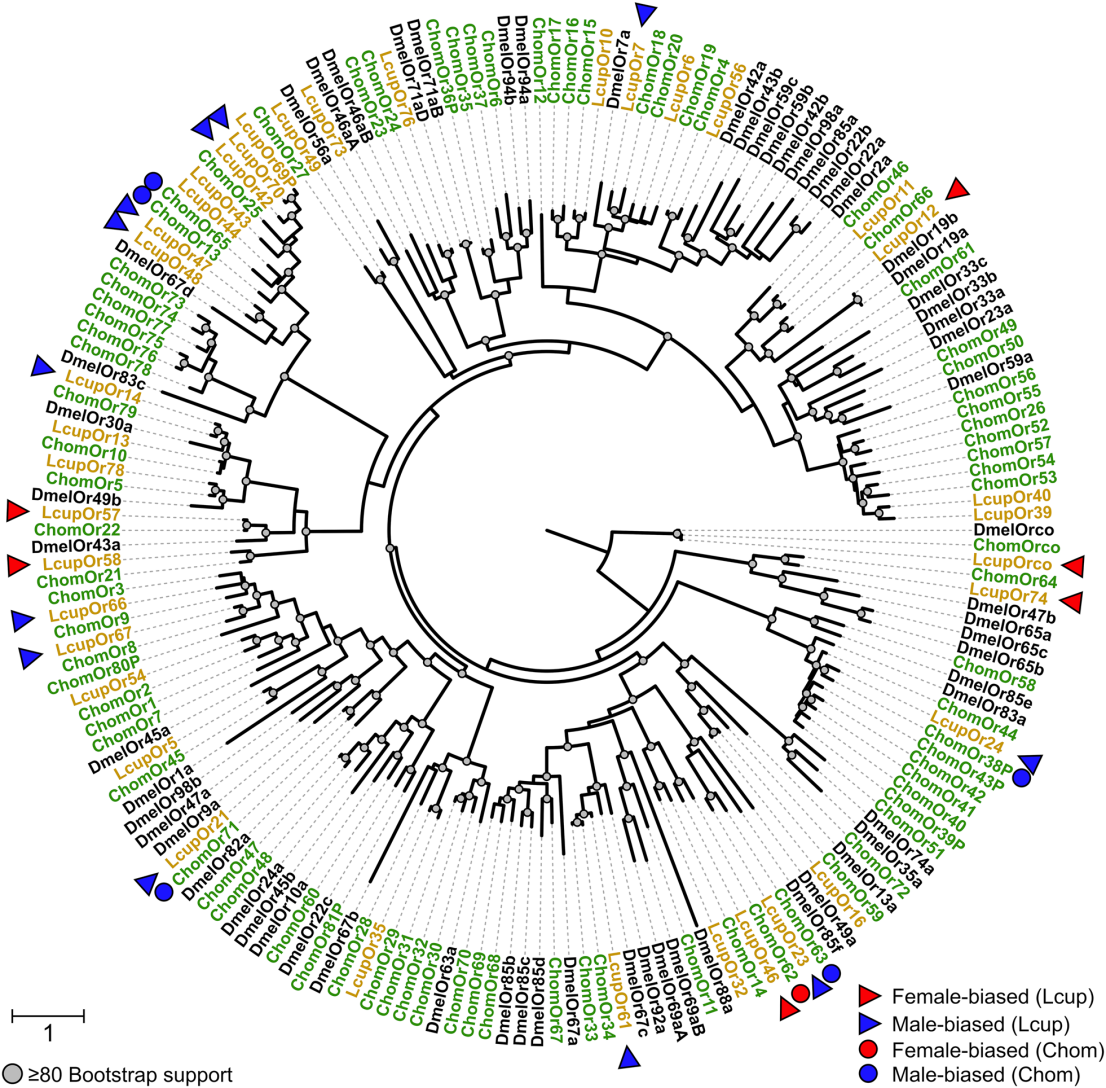
Phylogenetic analysis of odorant receptors (ORs). Three Diptera species were used for the phylogenetic analysis as follows: *Lucilia cuprina* (Lcup, gold), *Cochliomyia hominivorax* (Chom, green), *Drosophila melanogaster* (Dmel, black). Female- and male-biased ORs were detailed with red and blue colors, respectively. Sex-biased ORs in *L. cuprina* and *C. hominivorax* orthologs were detailed with triangles and circles, respectively. Nodes with a bootstrap supporting value ≥ 80 are indicated with a circle

Phylogenic analyses found that all six female-biased ORs were in different clades (Fig. 5). The ortholog of LcupOR46 was the only receptor upregulated in the female antennae of *C. hominivorax* [29]. LcupOR57 was another female-biased receptor, which is an orthologous sequence of DmelOR43a (Fig. 5). DmelOR43a was associated with the detection of benzaldehyde, benzyl alcohol, cyclohexanol and cyclohexanone [77], which are associated with vertebrate carrion [60, 78]. Six of the 12 male-biased ORs were clustered into three clades (Fig. 5), and four of those receptors, LcupOR42, 47, 48 and 70, were grouped into two clusters, representing a potential clade expansion in blowflies (Fig. 5). The same clade includes DmelOR67d (Fig. 5), which is an OR associated with pheromone detection and male courtship behavior in *D. melanogaster* [79]. Moreover, orthologs of seven of the male-biased ORs in *L. cuprina*, namely, 21, 23, 24, 42, 47, 48 and 70, were also male biased in the antenna of *C. hominivorax* [29].

Six of the IRs were male biased and four were female biased in *L. cuprina* antennae (Fig. S2 A). However, none of the orthologs of these transcripts were sex-biased in *C. hominivorax* [29], and the sex-biased IRs did not cluster into the same clade (Fig. S2 A). Two of female-biased IRs, LcupIR8a and 76b, are orthologs of IR coreceptors (*IRcos*) (Table S6). In *D. melanogaster*, it was observed that Dmel76b was essential for monovalent salt taste [80]. Interestingly, Rice et al. found that there is a high response *L. cuprina* females to monovalent cations, dependent on concentration [81]. The *D. melanogaster* ortholog of one of the other female-biased IRs, 64a, forms a complex with the IRco8a to form a single receptor that is associated with acid sensing [82]. The *D. melanogaster* orthologs of the male-biased LcupIR75b-1, 75d-1 and 92a are co-expressed in the same sensillum with an ammonium transporter (AMT) [38], suggesting a role in ammonia detection. Only one GR, LcupGR4, was sex-biased with higher expression in male antennae (Fig. S2 B).

**Fig. 6:**
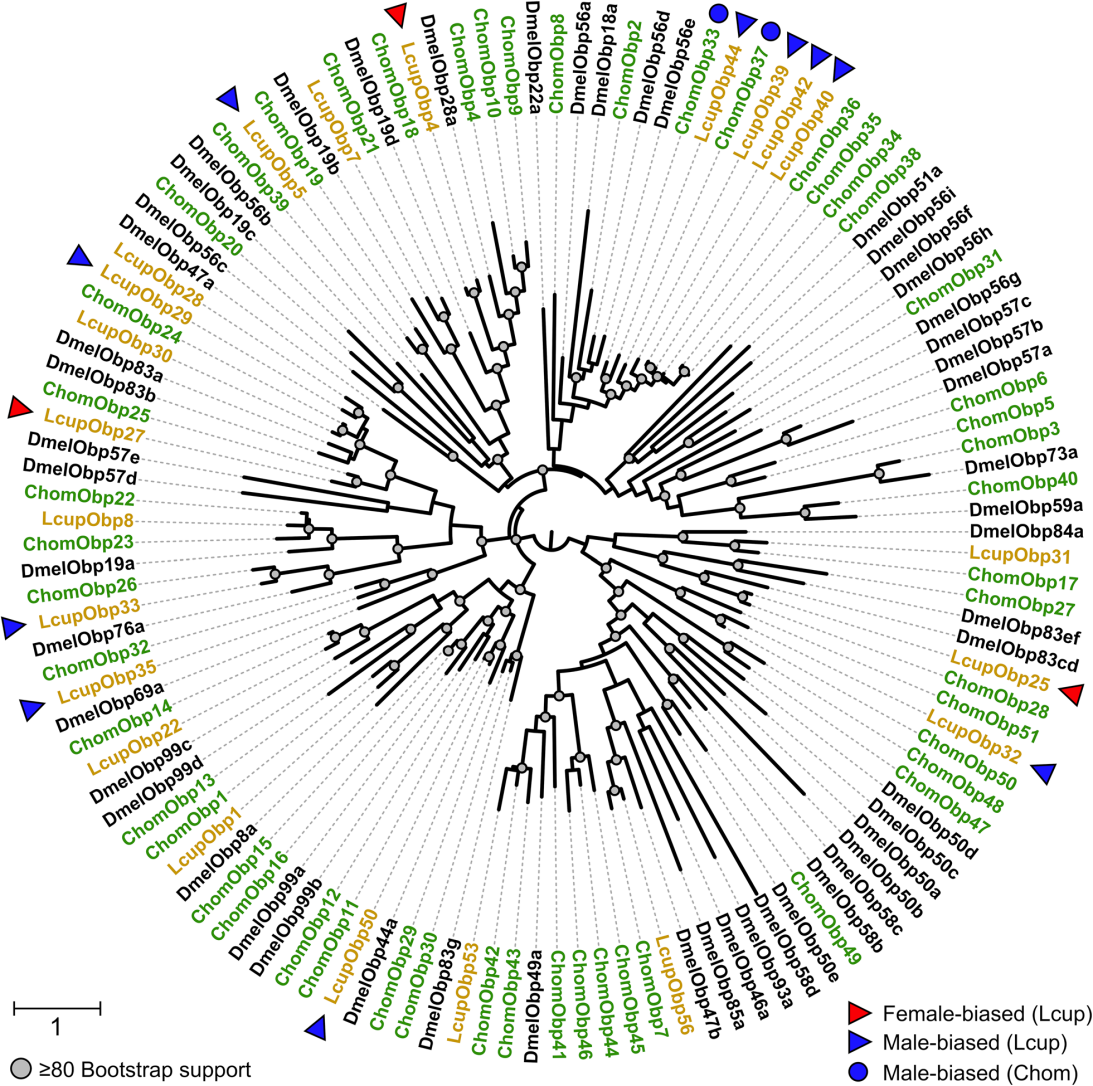
Phylogenetic analysis of odorant-binding proteins (OBPs). Three Diptera species were used for the phylogenetic analysis as follows: *Lucilia cuprina* (Lcup, gold), *Cochliomyia hominivorax* (Chom, green), *Drosophila melanogaster* (Dmel, black). Female- and male-biased OBPs were detailed with red and blue colors, respectively. Receptors biased in *L. cuprina* and *C. hominivorax* were detailed with triangles and circles, respectively. Nodes with a bootstrap supporting value > 80 were detailed

Of the *L. cuprina* female-biased OBPs, none were also female-biased in *C. hominivorax* [29]. LcupOBP4 was the most highly expressed transcript in the antenna of females (Table S3: D). The ortholog of LcupOBP4 in *D. melanogaster*, DmelOBP28a, was associated with butyric acid, 1-octanol and 2-pentanol detection, among other compounds [83], and all these compounds were associated with vertebrate carrion [4, 60]. Four of the male-biased antennal OBPs in *L. cuprina*, as follows, LcupOBP39, 40, 42 and 44, were clustered into the same clade (Fig. 6). Orthologs of these OBPs were also male biased in *C. hominivorax* [29]. These male-biased OBPs share a clade with DmelOBP56a (Fig. 6), which is associated with fatty acid binding in *D. melanogaster* [84].

Regarding other receptor gene families, we found two AMTs, four CD36/SNMPs and three CSPs differentially expressed between sexes (Fig. 4). The *L. cuprina* ortholog of ammonia transporter rhesus 50 (AMT_Rh50) was female-biased (Table S6: B). This receptor was associated with ammonia sensing in *D. melanogaster* [37, 38]. Ammonia-rich compounds (such as urine) were identified as important attractants for *L. cuprina* and *L. sericata* and stimulants for oviposition [16]. In sheep, females of these species lay eggs predominantly in the moist areas surrounding the perineal region of the animals, on the wool and skin in contact with feces and urine [16]; volatile compounds released by them have been shown to be attractive to the same species [64]. Consequently, AMT_Rh50 could have a relevant role in host detection by the females. In addition, AMT 2 was highly expressed in the antennae of both sexes and male biased (Table S6: B). In *Aedes aegypti*, two AMTs were associated with ammonia sensing and detoxification among other processes [85, 86]. These receptors were detected in several tissues including the antennae and reproductive organs, and one of them was essential for male mating success [85, 86]. Regarding CD36/SNMPs, in *D. melanogaster* members of this family have important roles in pheromone detection [45] and possibly could play a similar role in *L. cuprina*. One of the CSP transcripts was highly expressed in both sexes and female biased (Table S6: D). Interestingly, in *Bradysia odoriphaga* Winnertz, 1867 (Diptera: Sciaridae), a CSP was associated with detection of sulfur-rich compounds [87], which are common attractants for blowflies [16]. Two TRPs were male biased (Table S6: K); however, the expression of these receptors was very low. These receptors are associated with the detection of xenobiotic compounds [42], among other functions. Therefore, they could play a secondary/modulatory role in the detection of those compounds in *L. cuprina*.

#### GO enrichment

**Fig. 7:**
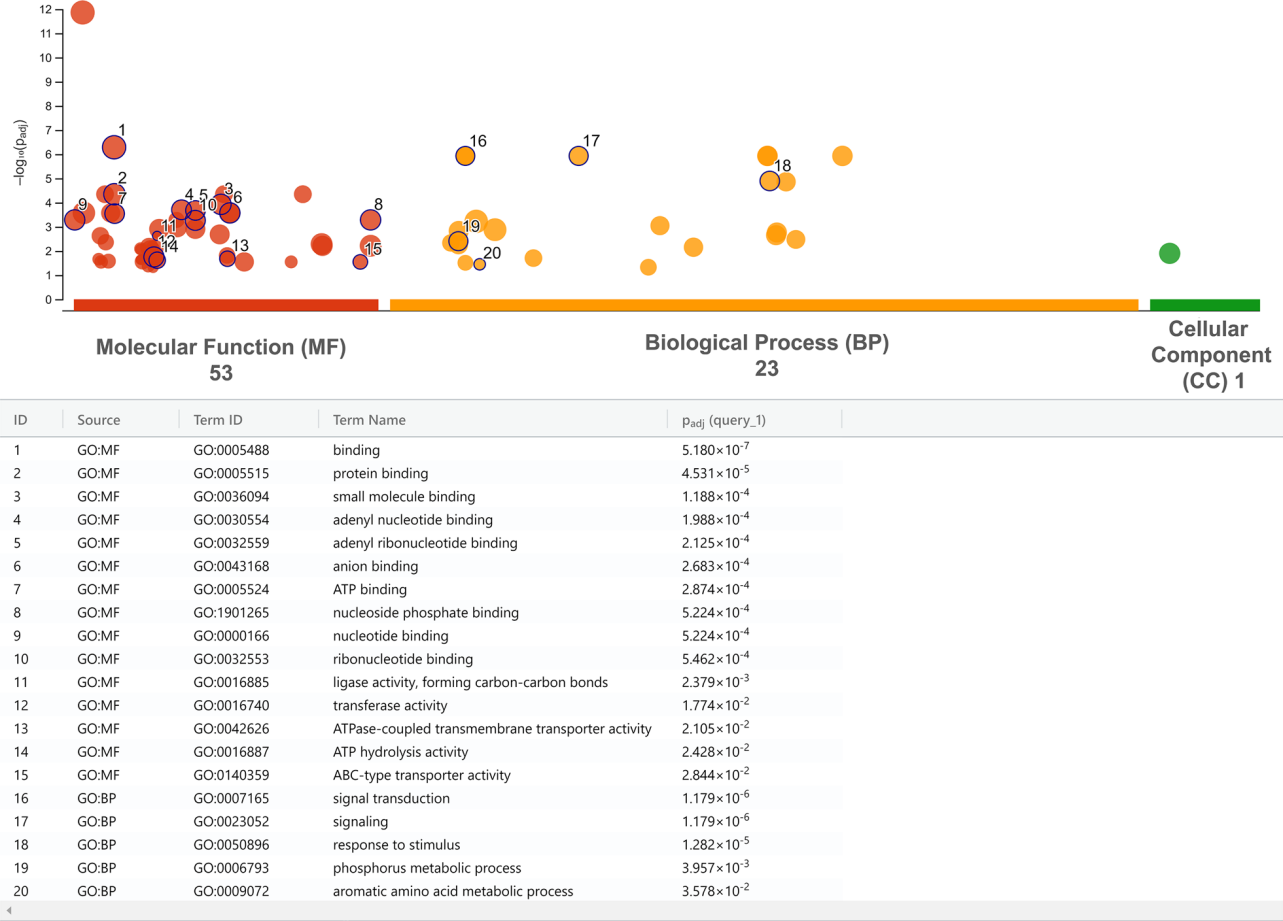
Gene Ontology (GO) terms enriched in attracted females compared to males. All GO terms enriched in attracted females vs. males were plotted as dots in the chart; however, only some of them, which were exclusively enriched in both types of females, were detailed with numbers and described below. The X-axis is the total number of GO terms enriched per main category (molecular function, biological process and cellular component), and the Y-axis is the statistical significance as the negative Log_10_ of the *P* adjusted value of the enriched GO terms. For all GO terms enriched for each group comparison, please refer to Table S8

A GO enrichment analysis was performed to gain a broader understanding of the DE transcripts between groups. Figure 7 shows GO terms enriched only in attracted females compared to males. We found many GO terms associated with ATP signaling among the top enriched GO terms (Fig. 7). These results are in line with the DGE analysis where ABC transporters showed a high number of upregulated receptors in the female antenna of *L. cuprina* (Fig. 4). Another GO term exclusively enriched in AF was the GO:0004930, which is linked to G protein-coupled receptor activity and related to neuropeptide signaling [88]. In line with this result, we found several transcripts that encode neuropeptides and their receptors (GPCRs) expressed in the antennae of both sexes. Similar neuropeptides and their receptors were identified in the antennae of other insects [89, 90]. The transcript encoding the ortholog of short neuropeptide F (sNPF) was the most highly expressed in all groups (Table S3: D). This peptide, together with the long neuropeptide F (NPF), was associated with molting, sleep, feeding, reproduction and immune response in insects [91]. Neuropeptides have been extensively studied in insects, but only a few works addressed neuropeptides in association with olfaction [91], and the role of many of them remains to be determined. In addition, while we found the transcripts for most neuropeptides were equally expressed between the sexes (Table S3: D), several neuropeptide receptors were upregulated in attracted females vs. males (Table S4: B) including diuretic hormone receptor, gonadotropin-releasing hormone receptor and neuropeptide Y receptor, for which the sNPF is the putative ligand (Table S4: B, yellow highlighted).

**Fig. 8:**
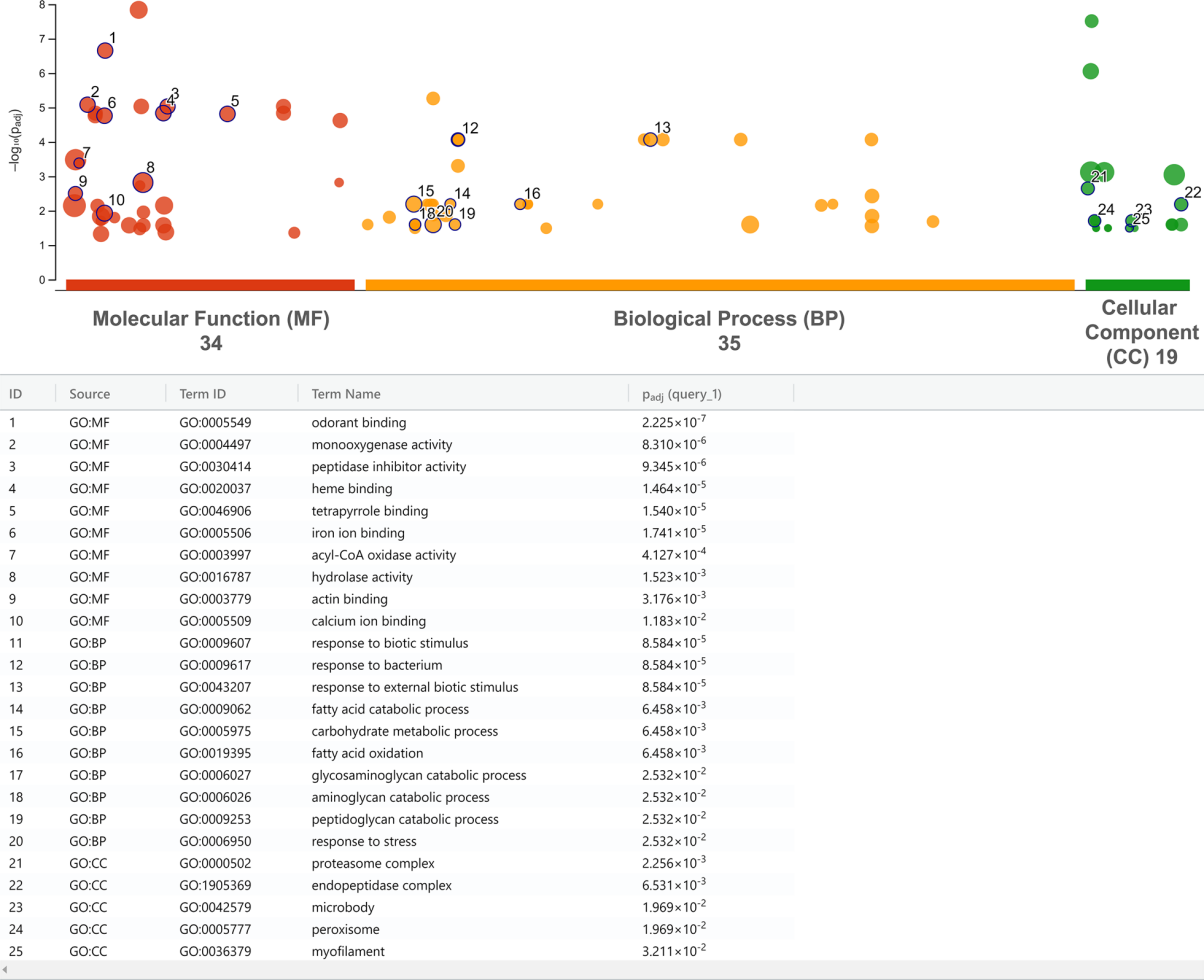
Gene Ontology (GO) terms enriched in males compared to attracted females. All GO terms enriched in males vs. attracted females were plotted as dots in the chart; however, only some of them which were exclusively enriched in males were detailed with numbers and described below. The X-axis is the total number of GO terms enriched per main category (molecular function, biological process and cellular component), and the Y-axis is the statistical significance as the negative Log_10_ of the *P* adjusted value of the enriched GO terms. For all GO terms enriched for each group comparison, please refer to Table S8

Figure 8 shows the GO terms enriched in males compared to attracted females. Some of the enriched GO terms were associated with odorant signaling (binding and degrading), such as GO:0005549, GO:0004497 and GO:0016787 (Fig. 8). In addition, we found several GO terms enriched in males associated with binding of different forms of iron, such as GO:0020037 and GO:0005506 (Fig. 8). In addition, several GO terms associated with the immune response were enriched in the male antennae, including GO:0009607 and GO:0009617 (Fig. 8). In *D. melanogaster*, iron and heme absorption is linked to several immune response processes, including the metal transporter Malvolio, iron-sulfur clusters and ABC transporters, among others [92]. Here, we found transcripts coding for these genes in the antennae of both sexes (Table S3: D), with some of them sex biased (Table S4: B-C). Furthermore, in *Locusta migratoria* Linnaeus, 1758 (Orthoptera: Acrididae), Zhang et al. [93] found an OBP linked to the immune response through the Toll-pathway innate immunity. In our study, the OBPs were the sensory gene family with the second highest number of male-biased sequences (Fig. 4) plus the higher average expression level among all sensory receptors (Table S6: H). Additionally, we found many transcripts associated with the Toll-pathway upregulated in the same group (Table S4: B-C). Moreover, Takeuchi et al. [94] found in *D. melanogaster* that the normal function and decay of a subset of olfactory receptor neurons is controlled by specific immune pathways, suggesting a close interaction between some genes associated with immune response and olfaction. Finally, for a listing of all enriched GO terms between all groups, refer to Table S8.

## Conclusions

More than a century of studies has extensively characterized the olfactory responses of *L. cuprina*; however, to our knowledge this is the first antennal transcriptome of *L. cuprina*. The main objective of the present work was the identification of sensory receptors potentially associated with host detection by females of *L. cuprina*. Our study has contributed to the characterization of differentially expressed sensory receptors between the antennae of both sexes and highlighted receptors potentially associated with host detection by females. We found good candidates to move forward with functional assays, such as the female-biased odorant receptor (LcupOR46), a female-biased ammonia transporter (AMT_Rh50) and an ABC transporter that was upregulated in the antennae of females attracted to rotting beef compared to non-attracted females (ABC G23.1). This is the first report suggesting a role for ABC transporters in *L. cuprina* olfaction. ABC transporters have been studied extensively in relation to resistance to insecticides and xenobiotic compounds in insects [95]; however, only recently have they been associated with olfaction [35]. Gene editing techniques will be used to silence the expression of each gene [96] in combination with behavioral assays to evaluate the role of these candidate genes in *L. cuprina* olfaction. Our long-term goal is to gain a better molecular understanding of how blowflies detect sulfur-rich compounds such as DMDS and ammonia, which are strong attractants.

### Supplementary Information

Supplementary material 1: Figure S1. The spatial olfactometer. A schematic of the olfactometer is shown in the upper left panel, and images show the main and collection chambers. The direction of airflow, beef sample location, fly release point, collection and main chambers are shown. The olfactometer scheme was originally made by Ann Carr and later modified by Juan P. Wulff.

Supplementary material 2: Table S1. Phylogenetic analysis. Sequences of *Cochliomyia hominivorax* and *Drosophila melanogaster* chemosensory proteins used for phylogenetic analysis. From tab A to D: *C. hominivorax* CDS and protein sequences of ORs, OBPs, IRs and GRs. In tabs E to H, *D. melanogaster* chemoreceptors names used for the same analysis are provided, together with the NCBI and FlyBase databases accession numbers.

Supplementary material 3: Table S2. Olfaction assay data. The table shows the number of flies trapped using fresh beef (collection chamber A), rotten beef (collection chamber B) and non-trapped (collected from the main chamber). In addition, the percentage of flies trapped relative to the totals are shown. The data from experiments with 30 or 50 released flies are listed separately.

Supplementary material 4: Table S3. *Lucilia cuprina* antennal transcriptome data. (A) number of raw, trimmed and mapped sequences per library; (B) assembly data; (C) raw counts per sequence and library for all transcripts, arranged by expression level from top (highest) to bottom (lowest); (D) TPM values per loci all transcripts for each replicate, and group averages highlighted in bold; (E) TPM expression per group for all transcripts after removing transcripts showing a TMP value < 5. (F) The sex-biased genes discussed in the manuscript are highlighted in bold. Abbreviations: TPM: transcript per million, AF: attracted female antennae, NF: non-attracted female, M: male antennae, GC%: guanine + cytosine content in percentage, AVG: average.

Supplementary material 5: Table S4. Differentially expressed transcripts in the antennae of *Lucilia cuprina*. (A) Transcripts expressed at a significantly higher or lower level in attracted females relative to non-attracted females (AF vs. NF tab). (B) Transcripts expressed at a significantly higher or lower level in attracted females relative to males (AF vs M tab). (C) Transcripts expressed at a significantly higher or lower level in non-attracted females relative to males (NF vs M tab). DESeq2 was used to determine differentially expressed transcripts, using a *P* adjusted value < 0.05, and without fold-change (FC) threshold, being Log_2_FC < 0 considered downregulated and Log_2_FC > 0 upregulated. The number of down- and upregulated transcripts and genes (in parentheses), for each comparison is shown at the top left of each sheet. The differential expression is provided in Log_2_FC and percentage. Putative odorant degrading enzymes (ODEs) were noted in the last column of each table; (D) all differentially expressed transcripts (TPM > 0) were plotted using volcano plots as gray crosses. . Differentially expressed genes between the groups that are discussed in the manuscript are highlighted in bold.The X-axis and Y-axis represent Log_2_ fold-change (Log_2_FC) differences between the contrasted groups and statistical significance as the negative Log_10_ of the *P* adjusted value, respectively. Abbreviations: DOWN: downregulated, UP: upregulated, TPM: transcript per million, AF: attracted female antenna, NF: non-attracted female, M: male antenna.

Supplementary material 6: Table S5. Differential gene expression (DGE) analysis: separated by groups. Differential expressed transcripts in the antennae of *Lucilia cuprina* were separated among different comparisons to highlight those transcripts potentially more relevant within each group. Only those transcripts with a TPM value ≥ 5 are shown in this table: (A) all results were compiled to show the number of differentially expressed transcripts for each group comparison; (B) transcripts upregulated in AF vs. both NF and M; (C) transcripts upregulated in NF vs. both AF and M; (D) transcripts upregulated in M vs. both AF and NF; (E) transcripts upregulated in both AF and NF vs. M; (F) transcripts upregulated in both AF and M vs. NF; (G) transcripts upregulated in both NF and M vs. AF. Differential transcript expression between groups in Log_2_ fold-change (Log_2_FC) and TPM expression (per group and on average) is provided for each sequence. Differentially expressed genes between the groups discussed in the manuscript are highlighted in bold. Abbreviations: TPM: transcript per million, AF: attracted female antenna, NF: non-attracted female, M: male antenna.

Supplementary material 7: Table S6. Transcript levels for sensory receptors identified in the antennae of *Lucilia cuprina* showing a TPM expression value ≥ 5. The average TPM level per group (AF, NF or M) and overall average is provided for each locus. Those differentially expressed transcripts between groups were noted, and the differential expressions were presented in Log_2_ fold-change (Log_2_FC). Abbreviations: ABC: ATP-binding cassette transporter, AMT: ammonium transporter receptor, CD36/SNMP: CD36 and sensory neuron membrane protein family, CSP: chemosensory protein, GR: gustatory receptor, IR: ionotropic receptor, OBP: odorant-binding protein, OR: odorant receptor, PPK: *pickpocket* receptor, TRP: transient potential receptor channel, AF: attracted female, NF: non-attracted female, M: male, TPM: transcript per million, AVG: average

Supplementary material 8: Table S7. Manually curated sensory receptor sequences. Only those sequences of odorant, gustatory and ionotropic receptors and odorant-binding proteins, showing a TMP expression value ≥ 5 (on average for all groups) in the antenna of *Lucilia cuprina* were curated. The first column of the table includes the name added to each receptor (gene column).

Supplementary material 9: Figure S2. Phylogenetic analysis of ionotropic receptors (A) and gustatory receptors (B). Three Diptera species were used for the phylogenetic analysis as follows: *Lucilia cuprina* (Lcup, gold), *Cochliomyia hominivorax* (Chom, green), *Drosophila melanogaster* (Dmel, black). Female- and male-biased IRs and GRs were detailed with red and blue colors, respectively. Receptors biased in *L. cuprina* and *C. hominivorax* were detailed with triangles and circles, respectively. Nodes with a bootstrap supporting value > 80 were detailed. Abbreviations: GR: gustatory receptor, IR: ionotropic receptor.

Supplementary material 10: Table S8. Gene Ontology (GO) enrichment analysis. GO terms enriched for each pairwise group comparison: (A) AF vs. NF (no GO terms enriched); (B) NF vs. AF; (C) AF vs. M; (D) M vs. AF; (E) NF vs. M; (G) M vs. NF. GO terms with a *P* adjusted value > 0.05 were considered enriched. GO terms enriched in both types of females, AF vs. M and NF vs. M (sheets C and E), were bold highlighted. Furthermore, GO terms enriched in males vs. both types of females were also bold highlighted (sheets D and F). Columns (from a to i) within each tab include all the data analysis as follows: (a) parental GO-term; (b) specific GO term name; (c) specific GO-term ID; (d) adjusted* P* value that determined if a GO term was enriched; (e) negative -LOG_10_ of adjusted *P* value used for plotting; (f) number of genes associated with the given specific GO-term; (g) LOCI used as query; (h) number of domains in the database; (i) loci associated with the specific given GO term. Abbreviations: AF: attracted female antenna, NF: non-attracted female, M: male antenna.

## Data Availability

All relevant data are in the manuscript. Raw sequences were uploaded to the NCBI database under Bioproject PRJNA1092525.

## Abbreviations

AF: Attracted females
ABC: ATP-binding cassette transporter
AMT: Ammonium transporter
BLAST: Basic local alignment search tool
CDS: Coding sequence
CFM: Cubic foot per minute
CNS: Central nervous system
CD36/SNMP: CD36 and sensory neuron membrane proteins
cDNA: Complementary DNA
COX: Cytochrome c oxidase
CO_2_: Carbon dioxide
CSP: Chemosensory protein
DE: Differential expressed
DEPP: Department of Entomology and Plant Pathology
DGE: Differential gene expression
DNA: Deoxyribonucleic acid
dNTP: Deoxynucleotide triphosphate
DMDS: Dimethyl disulfide
DMTS: Dimethyl trisulfide
FC: Fold-Change
g: Gram
GC/MS: Gas chromatography/mass spectrometry
GO: Gene Ontology
GPCR: G protein-coupled receptor
GR: Gustatory receptor
h: Hour
H_2_S: Hydrogen sulfide
IPM: Integrated pest management
IR: Ionotropic receptor
K-ATP: ATP-dependent potassium channels
M: Males
ML/NPC2: Myeloid lipid-recognition (ML) and Niemann-Pick C2 disease proteins
mRNA: Messenger RNA
NCSU: North Carolina State University
NCBI: National Center for Biotechnology Information
NF: Non-attracted females
NPF: Long neuropeptide F
OBP: Odorant-binding protein
ODE: Odorant-degrading enzyme
OR: Odorant receptor
Orco: Odorant receptor coreceptor
OSNs: Olfactory sensory neurons
PE: Paired end
PPK: Pickpocket receptor
RH: Relative humidity
RNA: Ribonucleic acid
RNA-Seq: RNA sequencing
RT: Room temperature
SEM: Standard error of the mean
SIT: Sterile insect technique
sNPF: Short neuropeptide F
SRB1: Scavenger receptor class B type I
SURx: Sulfonylurea receptor
TRP: Transient receptor potential
VOC: Volatile organic compounds
*wt*: Wildtype
