# Supplementary figures and images for: Antennal transcriptome analysis reveals sensory receptors potentially associated with host detection in the livestock pest Lucilia cuprina
Source: Parasit Vectors. 2024 Jul 18;17:308. doi: 10.1186/s13071-024-06391-6 (PMC11256703; doi:10.1186/s13071-024-06391-6)

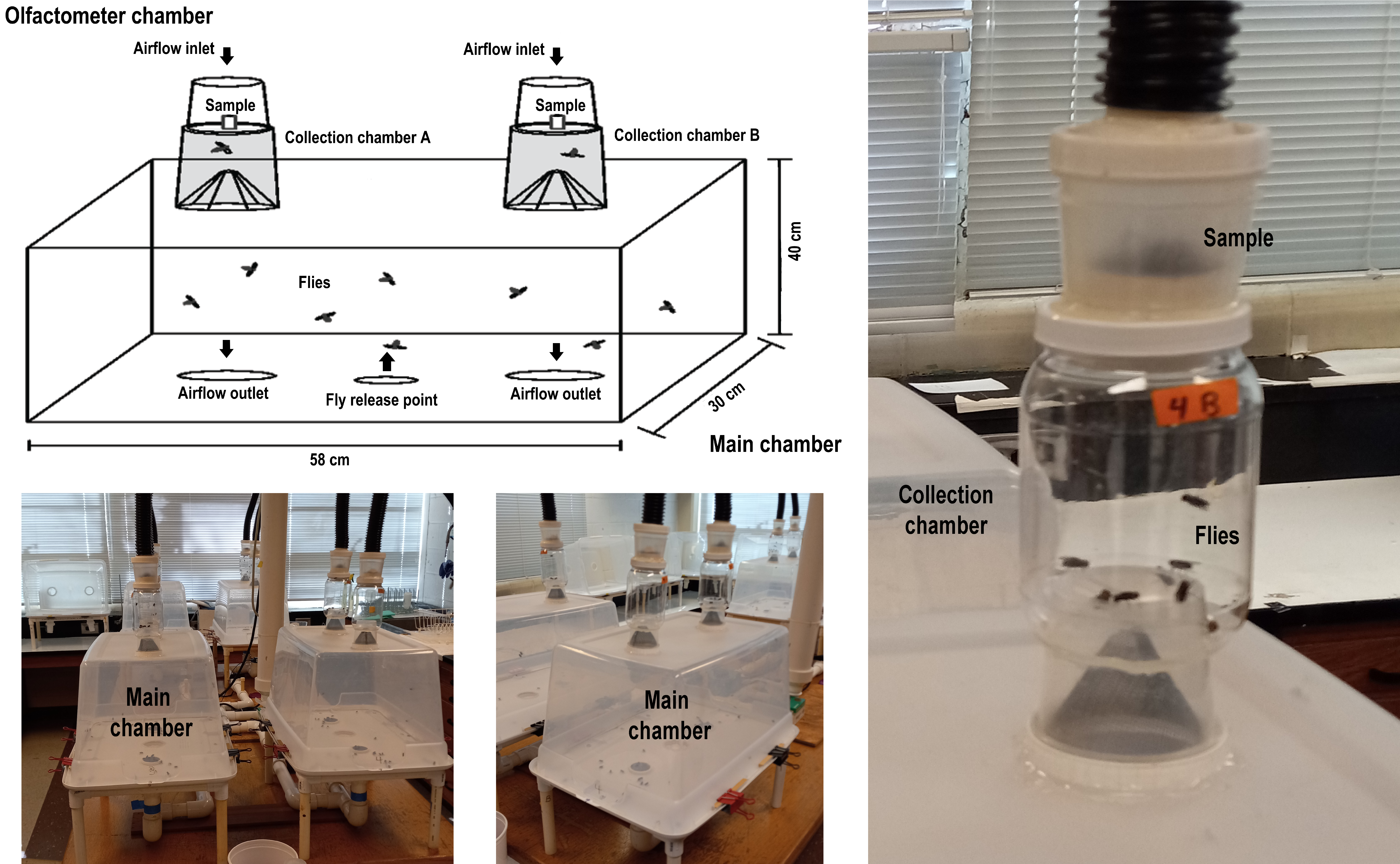

Supplement: Supplementary file 1 — Supplementary material 1: Figure S1. The spatial olfactometer. A schematic of the olfactometer is shown in the upper left panel, and images show the main and collection chambers. The direction of airflow, beef sample location, fly release point, collection and main chambers are shown. The olfactometer scheme was originally made by Ann Carr and later modified by Juan P. Wulff. [file 13071_2024_6391_MOESM1_ESM.png]

### A. Ionotropic receptors

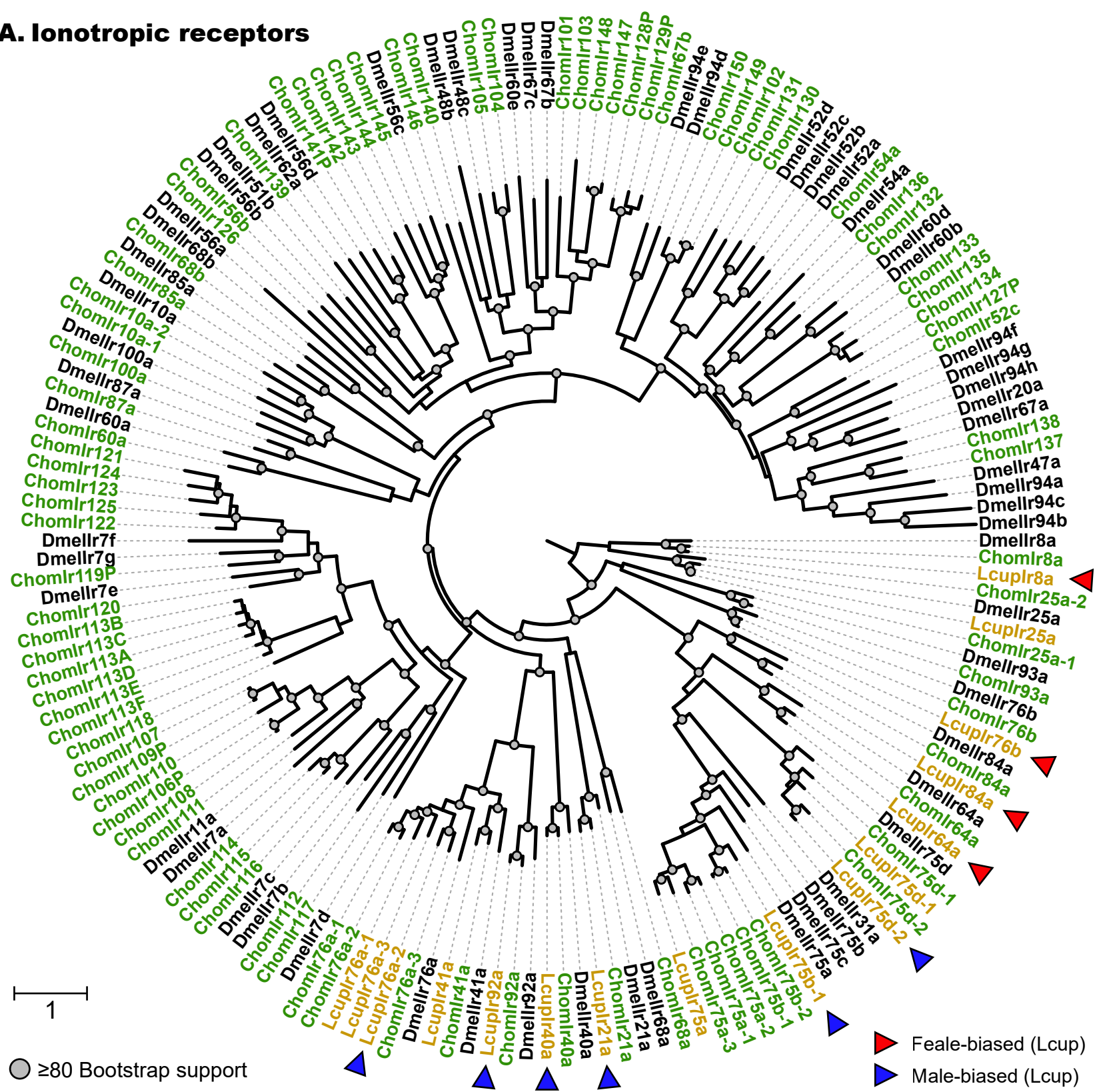

## B. Gustatory receptors

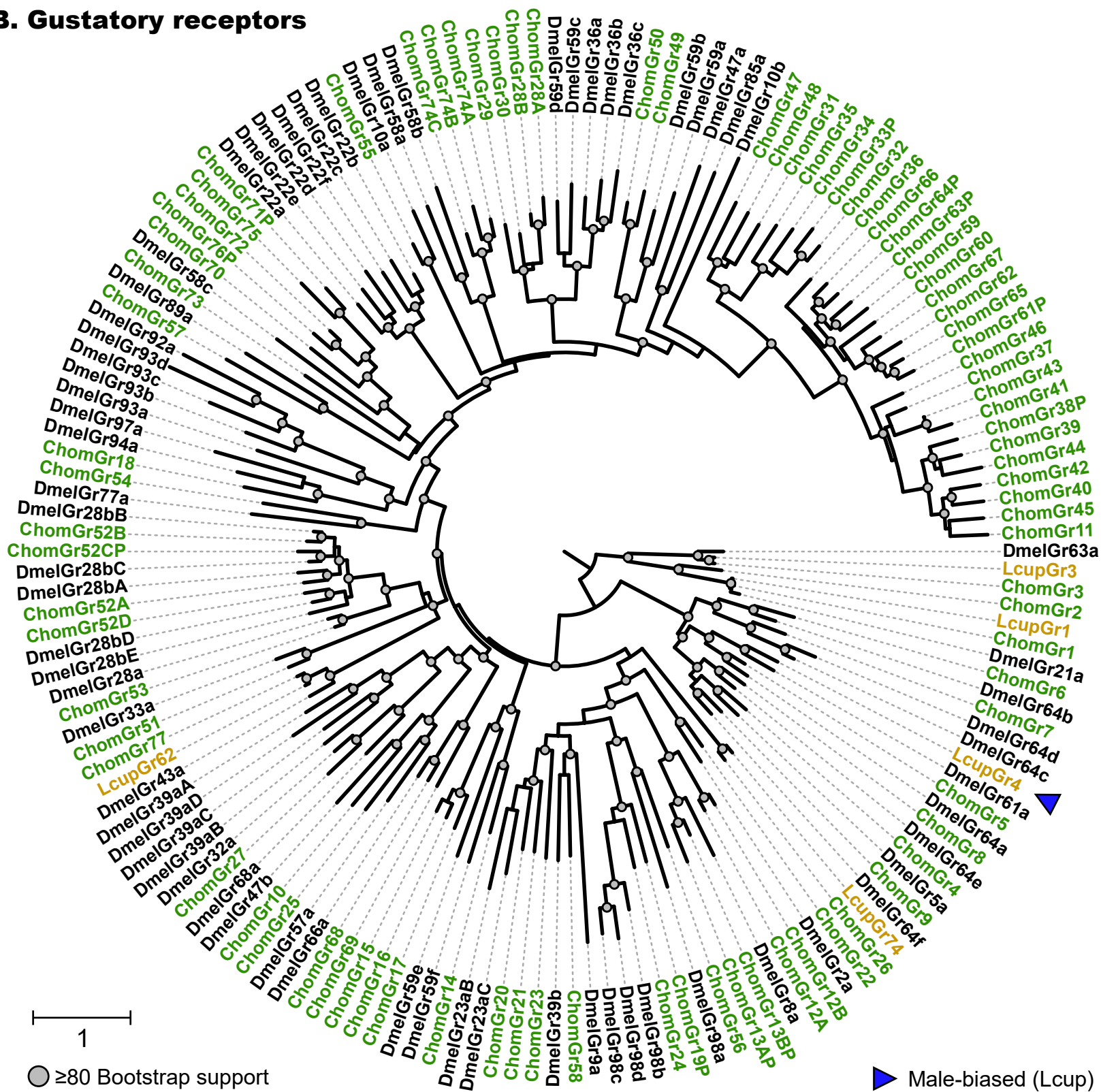

Supplement: Supplementary file 9 — Supplementary material 9: Figure S2. Phylogenetic analysis of ionotropic receptors (A) and gustatory receptors (B). Three Diptera species were used for the phylogenetic analysis as follows: Lucilia cuprina (Lcup, gold), Cochliomyia hominivorax (Chom, green), Drosophila melanogaster (Dmel, black). Female- and male-biased IRs and GRs were detailed with red and blue colors, respectively. Receptors biased in L. cuprina and C. hominivorax were detailed with triangles and circles, respectively. Nodes with a bootstrap supporting value > 80 were detailed. Abbreviations: GR: gustatory receptor, IR: ionotropic receptor. [file 13071_2024_6391_MOESM9_ESM.pdf]
